# Serum 25-Hydroxyvitamin D Concentrations and Atopic Dermatitis in Early Childhood: Findings from the Japan Environment and Children’s Study

**DOI:** 10.3390/nu13082761

**Published:** 2021-08-12

**Authors:** Limin Yang, Miori Sato, Mayako Saito-Abe, Minaho Nishizato, Hidetoshi Mezawa, Kiwako Yamamoto-Hanada, Yukihiro Ohya, The Japan Environment and Children’s Study (JECS) Group

**Affiliations:** 1 Allergy Center, National Center for Child Health and Development, Tokyo 157-8535, Japan; sato-m@ncchd.go.jp (M.S.); saito-myk@ncchd.go.jp (M.S.-A.); nishizato-m@ncchd.go.jp (M.N.); mezawa-h@ncchd.go.jp (H.M.); yamamoto-k@ncchd.go.jp (K.Y.-H.); ohya-y@ncchd.go.jp (Y.O.); 2 Medical Support Center for the Japan Environment and Children’s Study, National Research Institute for Child Health and Development, Tokyo 157-0074, Japan

**Keywords:** 25-hydroxyvitamin D, vitamin D, deficiency, insufficiency, children, atopic dermatitis

## Abstract

Vitamin D (VitD) may affect immune system modulation and result in the development of atopic dermatitis (AD). However, published findings have remained controversial. We investigated the association between early-life 25-hydroxyvitamin D (25(OH)D) levels and AD risk at childhood with a birth cohort. The data were obtained from “the Japan Environment and Children’s Study (JECS)” and “the Sub-Cohort study of JECS” performed with children aged 2 years. “Liquid chromatography-tandem mass spectrometry” was used to measure VitD. The information on AD was obtained from parents’ answers to a questionnaire when their children were aged 3 years. In order to explain the seasonal effects on VitD levels, a deseasonalized continuous variable was further calculated. The logistic regression models were fitted to evaluate the effect of VitD on childhood AD. The study included 4378 children with complete data on VitD and AD. The results from models indicated that low VitD at 2 years was not a risk factor for the development of AD at 3 years, after adjusting for potential confounders. Moreover, there was no U-shape relationship between deseasonalized VitD and childhood AD. Overall, early-life 25(OH)D levels were not link to the increased risk of developing childhood AD.

## 1. Introduction

Atopic dermatitis (AD), also named atopic eczema, is the one of the most common chronic inflammatory skin disorders during childhood [1,2], with an estimated prevalence of around 10–30% among children. The development of AD has still remained incompletely understood, with studies showing the influence of genetic, environmental (e.g., pollen, house dust, and mites), and dietary factors [3,4].

Vitamin D (VitD) can be formed in the human skin through sunlight exposure or obtained from dietary intake [5,6,7]. It is essential for the maintenance of bone health [6,8], regulating calcium and phosphate metabolism [6,9]. Moreover, VitD is crucial in normal cutaneous physiology and immune response [10,11] and appears to strengthen the epidermal barrier [10,12]. Studies also revealed that VitD promotes human cathelicidin generation, which has been considered insufficient in AD [10,13] and improves innate immunity. Given that the mechanism of development of AD relates to skin barrier dysfunction and immune response dysregulation [14,15], VitD might be related thereto. Epidemiological studies have also shown evidence with regard to VitD and the development of atopic eczema [16]. For instance, a trial performed among Mongolian children aged 2–17 years found that VitD supplementation appeared to improve AD among children [17]. Another study among adults also indicated that obese populations with VitD deficiency had higher risk for AD compared to those without VitD deficiency [18].

25-Hydroxyvitamin D (25(OH)D) is a stable VitD metabolite in the circulation [19]. Measurements of 25(OH)D have been considered to be a best approach for determining vitamin D status in the body [20].

Therefore, we designed this research study in order to address the issue involving early life 25(OH)D levels and childhood AD by using data from a large cohort dataset. Accordingly, we hypothesized that either low or high VitD levels would increase the odds of developing AD among young population.

## 2. Methods

### 2.1. Population and Variables

The participants included herein were recruited from a large birth cohort, namely “The Japan Environment and Children’s Study (JECS)”, with 104,062 fetal records [21,22]. The JECS primarily aimed to evaluate the relationship between “environmental exposure and children’s health and development”, with the design having been detailed elsewhere [21,23,24]. Briefly, JECS is an ongoing 13 year follow-up study. Pregnant women were recruited during 2011–2014 [25,26]. In addition to the main study, JECS also includes a Sub-Cohort Study with around 5000 participants [27].

The JECS protocol was reviewed and approved by “The Ministry of the Environment’s Institutional Review Board on Epidemiological Studies” and “The Ethics Committees of all participating institutions (Ethical Number: No.100910001)” [26,28,29]. Data used for analysis were obtained from the jecs-ta-20190930-qsn and jecs-ta-20190930-mdv datasets [26].

**Figure 1:**
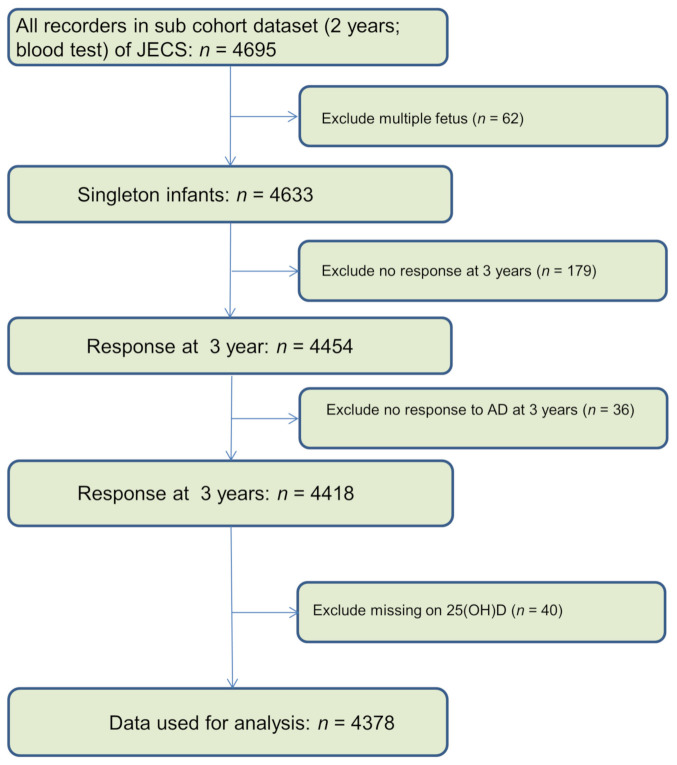
Flow chart of data selection.

The current study established a cohort to explore the relationship between vitamin D at 2 years and AD at 3 years. Participants with unknown pregnancy outcomes were excluded from analysis. Only singleton live born offspring who had complete data on VitD and the parents-reported AD at 3 years were included herein. Details regarding data selection are outlined in Figure 1.

Plasm 25(OH)D levels were measured using blood specimens collected from the participating children at 2 years. The total amount of blood collected was 4 mL. “Liquid chromatography-tandem mass spectrometry (LC-MS/MS)” was used to measure 25(OH)D_3_ (cholecalciferol) and 25(OH)D_2_ (ergocalciferol) levels. Plasm 25(OH)D in this study amounts to the sum of ergocalciferol and cholecalciferol.

In order to explain the seasonal effects on VitD levels, a deseasonalized continuous variable was further calculated [30].

First, we built a “sinusoidal model” [30] as follows:

(1) $$25\left(\mathrm{OH}\right)D\;\mathrm{level}=\beta_{0}+\beta_{1}\sin\frac{2\pi m}{12}+\beta_{2}\cos\frac{2\pi m}{12}$$

where *m* indicated the timing of the test (month). Thereafter, residuals of the regression model were calculated for each individual. A seasonally adjusted value was the sum of the residual and overall mean.

Information on AD was obtained from the parents’ answers to the questionnaire when their child turned 3 years old, which was based on a “modified Japanese-translated version of the International Study of Asthma and Allergies in Childhood (ISAAC)” and “was validated based on the ISAAC protocol for 6- to 7-year-old children” [26,31,32,33]. The detailed definition of AD has been described elsewhere [26].

The following were considered as potential confounders based on the previous studies: gender (boys/girls); z scores of body mass index (BMI) at 2 years; mother’ age (<35 years/≥35 years); history of abnormality of pregnancy (no/yes); maternal smoking (no/yes); paternal smoking (no/yes); maternal history of AD (no/yes); maternal level of education (low/normal of high); family income; pet keeping (no/yes); pregnancy complications (no/yes); obstetric complications (no/yes); gestational age (<37 weeks/≥37 weeks); BMI before pregnancy (<25/≥25); breast feeding (no/yes); kindergarten (no/yes); and parity (nulliparous/multipara). Variables (gender and parity) were transcribed from medical records after delivery. Others were transcribed from the questionnaire during pregnancy and after delivery.

### 2.2. Statistical Analysis

The generalized linear models with the logit function (logistic regression models) were fitted for AD at age 3 years. We fitted two models with different confounding factors. The first model only adjusted for gender as the confounder, while the second model adjusted all the confounders mentioned above and the season in which the blood tests were conducted. Odds ratios (ORs) indicate the odds of reporting AD versus no AD in children. We calculated the variance inflation factor in order to determine the collinearity in the models. The Wald tests were used to test the significance of the associations.

VitD at 2 years was grouped as deficiency, insufficiency and normal groups by using 20 and 30 ng/mL as the cut points. The deseasonalized VitD in the models was divided into five categories according to quantile (using the middle group as reference).

We assumed that the mechanisms of missingness were “missing at random” and used multiple imputation (MI) to deal with missing data [26,34]. A total of 20 data sets were generated and used for calculating pooled ORs. The variables in imputation models included all independent variables in the GLM when fitting AD.

Multiple test adjustment for p values was not conducted because of “the exploratory nature” of our report [26].

Logistic models with season adjusted values of VitD as continuous variables were further developed to explore nonlinear relationship between early life vitamin D and AD. “Restricted cubic splines” were used for the continuous variables in the models. We then plotted the graph with the *x*-axis as the deseasonalized VitD and the *y*-axis as the ORs (95% CI) of AD. In addition, the analysis was repeated by including only those with complete data on AD and all the independent variables. We also assessed the modified effect of “maternal history of AD”. Finally, subgroup analysis was performed. The model was rerun among children without AD at 1 and 2 years.

All calculations were carried out with R software (version 4.0.3) [35].

## 3. Results

### 3.1. Participant Characteristics

The distribution of covariates among those included and excluded from analysis is summarized in Table S1. There were no significant differences between the two groups. The prevalence of AD was 13.3% in children aged 3 years. The median (interquartile range) of VitD was 24.6 (8.5) ng/mL.

### 3.2. VitD and Childhood Atopic Dermatitis

The odds ratios for serum 25(OH)D levels from the logistic models are listed in Table 1 After adjusting for confounders, VitD deficiency (OR: 0.97; 95% CI: (0.74–1.27)) or insufficiency (OR: 0.92; 95% CI: (0.73–1.14)) did not increase the possibility of childhood AD. Compared to reference group (category 3), low (category 1 and 2) or high levels (category 4 and 5) of deseasonalized VitD showed no marked effect on childhood AD (Table 2).

### 3.3. Sensitivity Analyses

**Figure 2:**
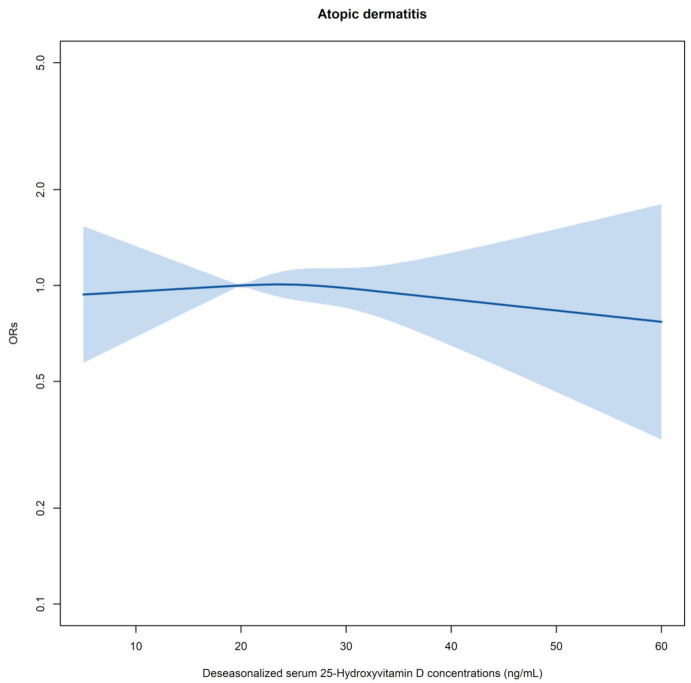
Summary of logistic regression models for estimating the relationship between deseasonalized vitamin D and atopic dermatitis in early childhood. Deseasonalized vitamin D was treated as a continuous variable. Model adjusted for all confounders listed in Table S1. Reference for ORs: 20 ng/mL. Shaded bands: 95% confidence limits for ORs. ORs: odds ratios.

There was no modified effect of “maternal history of AD”. Stratified analysis by this variable showed similar non-significant ORs (Table S2). The results from models fitting the complete data showed very similar results (data not shown). When analysis was limited to those without AD at 1 and 2 years, no obvious significant association were observed (Table 2). After further adjusting for season in the models, deseasonalized VitD showed no marked effect on AD (results not shown). No significant U-shape relationship was found when the variable deseasonalized VitD was placed into the models as a continuous variable (Figure 2).

## 4. Discussion

The current study found no evidence to support the hypothesis that lower VitD levels increase the odds of developing AD during early childhood. Moreover, VitD level at 2 years was unable to predict AD at 3 years among children without AD at 1–2 years. This has been the first effort to evaluate this association with a representative Japanese cohort.

To date, limited reports have been made available on the issue involving early life VitD and childhood AD. Some published reports have suggested that maternal VitD measured before pregnancy, during pregnancy, or at delivery were not associated with increased odds in the development childhood AD [1,11,36,37]. A cohort study in Norway found that vitamin D in children aged 0–2 years was not significantly related to caregiver-reported atopic eczema [38]. Our results are also similar to some published cross-sectional studies. For example, Cairncross et al. demonstrated that 25 (OH)D concentrations did not change the odds of eczema in children aged 2–5 years in New Zealand [39]. A recently published cross-sectional study in Germany by Reinehr et al. compared VitD levels between children with and without AD and found no significant differences between the two groups [40].

Other studies, however, have published conflicting conclusions, with increased eczema risk being associated with low/high VitD levels. Accordingly, Heimbeck et al. found that low VitD status is inversely related to eczema based on evidence from children aged 1–17 in a cross-sectional survey in Germany [41]. Meanwhile, a study performed in the UK by Gale et al. indicated that children whose mothers had 25(OH)D concentrations higher than 75 nmol/L during pregnancy were more likely to have atopic eczema at age 9 months compared to those exposed to normal VitD levels [42]. By contrast, Jone et al. found that infant with higher cord blood VitD had reduced odds of developing eczema at 6–12 months [43]. The current study did not find similar results. The differences in results involving this issue may have been attributed to differences in the study population and sample sizes, outcome definitions, and the time points at which 25(OH)D was measured. Further cohort studies are still needed in order to replicate and confirm our findings.

Although the aforementioned studies implied that lower VitD levels may promote increased or decreased AD in children, our findings did not observe any similar evidence in the younger Japanese population. Other environmental/genetic factors, such as filaggrin loss-of-function mutations, seem to be more important in explaining childhood AD [44]. UV-B is another important confounder when investigating whether VitD deficiency results in increased risk of childhood AD. Future studies are necessary to evaluate the effects of genetic and environmental factors on the development of AD among children [45].

Our study has some limitations. Firstly, information on AD was collected by parents by using questionnaires. For this reason, some misclassifications of AD could still exist due to the lack of validation from physical examination or medical records, which might have reduced the power of this study. However, the “ISAAC questionnaire” is commonly used in large epidemiological studies and has been proven to have good internal consistency [26]. Secondly, we could not adjust for some important confounder, such as sunlight exposure and supplementation intake, due to the lack of data in the JECS. Thirdly, VitD was measured only once. We do not think this could bias our results significantly. Saliba et al., who investigated the correlation between values measured 1 year apart in the same month of the year among participants who had not used supplements 6 months before blood sampling, reported a good consistency [46]. Finally, negative results could also be attributed to uninvestigated variables or other environmental and genetic confounders that have not yet been identified.

In conclusion, although the results from animal models and epidemiology studies imply a relationship between VitD and childhood AD, our findings suggest no such association among Japanese children aged 2 years. Further longitudinal studies investigating other environmental/genetic factors are still needed to confirm the results in this study. Moreover, future studies should focus on other active forms of VitD, such as 1,25-dihydroxyvitamin D, in order to evaluate this topic again.

## Figures and Tables

**Table 1:** Odds ratios from the logistic regression model for early life vitamin D levels and childhood atopic dermatitis.

25(OH)D (ng/mL)	Odds Ratios (95% CI) ^$^	Odds Ratios (95% CI) ^&^
ALL		
<20 vs. ≥30	1.06 (0.83–1.35)	0.97 (0.74–1.27)
≥20 and <30 vs. ≥30	0.96 (0.77–1.19)	0.92 (0.73–1.14)
Sub dataset ^#^		
<20 vs. ≥30	1.25 (0.79–1.98)	1.23 (0.75–2.03)
≥20 and <30 vs. ≥30	1.22 (0.82–1.83)	1.21 (0.80–1.83)

CI: confidential inference. ^#^ Analysis was limited to those without atopic dermatitis at 1 and 2 years. ^$^ Adjusted for gender as a confounder. ^&^ Adjusted for all confounders listed in Table S1.

**Table 2:** Odds ratios from the logistic regression model for deseasonalized vitamin D levels.

Deseasonalized 25(OH)D	ORs (95% CI) ^$^	ORs (95% CI) ^&^
All		
Deseasonalized 25(OH)D category 1 vs. 3	0.91 (0.69–1.20)	0.91 (0.69–1.21)
Deseasonalized 25(OH)D category 2 vs. 3	0.92 (0.70–1.21)	0.95 (0.72–1.26)
Deseasonalized 25(OH)D category 4 vs. 3	0.92 (0.70–1.21)	0.89 (0.67–1.17)
Deseasonalized 25(OH)D category 5 vs. 3	0.93 (0.71–1.22)	0.94 (0.71–1.23)
Sub dataset ^#^		
Deseasonalized 25(OH)D category 1 vs. 3	0.89 (0.57–1.41)	0.90 (0.57–1.42)
Deseasonalized 25(OH)D category 2 vs. 3	0.70 (0.43–1.14)	0.72 (0.44–1.17)
Deseasonalized 25(OH)D category 4 vs. 3	0.80 (0.51–1.28)	0.79 (0.50–1.27)
Deseasonalized 25(OH)D category 5 vs. 3	0.64 (0.39–1.05)	0.65 (0.40–1.07)

ORs: odds ratios; CI: confidential inference. ^#^ Analysis was limited to those without AD at 1 and 2 years. ^$^ Adjusted for gender as a confounder. ^&^ Adjusted for all confounders listed in Table S1.

## Data Availability

Data are not permitted for public deposition.

## Supplementary Materials

The following are available online at https://www.mdpi.com/article/10.3390/nu13082761/s1, Table S1: Distribution of covariates among those included and excluded from analysis according to the selection criteria, Table S2: Modified effect of maternal history of atopic dermatitis.

## Abbreviations

Vitamin D: VitD; JECS: Japan Environment and Children’s Study; ISAAC: International Study of Asthma and Allergies in Childhood; OR: odds ratio; CI: confidence interval; AD: atopic dermatitis.
