# Supplementary material for: Serum 25-Hydroxyvitamin D Concentrations and Atopic Dermatitis in Early Childhood: Findings from the Japan Environment and Children’s Study
Source: Nutrients. 2021 Aug 12;13(8):2761. doi: 10.3390/nu13082761 (PMC8401201; doi:10.3390/nu13082761)
Supplement: Supplementary file 1 [file nutrients-13-02761-s001.zip › nutrients-1306742-supplementary.pdf]

Table S1 Distribution of covariates among those included and excluded from analysis according to the selection criteria.

		Data used for analysis				Excluded data			
				95% CI				95% CI	
Variables	Category	n	%	Lower	Upper	n	%	Lower	Upper
Confounders									
History of abnormality of pregnancy	No	4092	93.5	92.7	94.2	72	94.7	86.4	98.3
	Yes	286	6.5	5.8	7.3	4	5.3	1.7	13.6
	Missing	0	-			0	-		
Maternal smoking	No	4209	96.7	96.1	97.2	72	94.7	86.4	98.3
	Yes	145	3.3	2.8	3.9	4	5.3	1.7	13.6
	Missing	24	-			0	-		
Paternal smoking	No	2564	59.4	57.9	60.9	41	53.9	42.2	65.3
	Yes	1752	40.6	39.1	42.1	35	46.1	34.7	57.8
	Missing	62	-			0	-		
Maternal history of atopic dermatitis	No	3624	82.8	81.6	83.9	62	81.6	70.7	89.2
	Yes	754	17.2	16.1	18.4	14	18.4	10.8	29.3
	Missing	0	-			0	-		
Maternal level of education	Normal or high	3034	69.6	68.2	71	48	63.2	51.3	73.7
	Low	1323	30.4	29	31.8	28	36.8	26.3	48.7
	Missing	21	-			0	-		
Income	≥4 million yen/per year	2656	63.4	62	64.9	46	62.2	50.1	73
	<4 million yen/per year	1530	36.6	35.1	38	28	37.8	27	49.9
	Missing	192	-			2	-		
Pet keeping	No	3418	78.4	77.2	79.7	60	78.9	67.8	87.1
	Yes	939	21.6	20.3	22.8	16	21.1	12.9	32.2

	Missing	21	-			0	-		
Pregnancy complications	No	3624	83.1	82	84.2	60	81.1	70	88.9
	Yes	735	16.9	15.8	18	14	18.9	11.1	30
	Missing	19	-			2	-		
Obstetric complications	No	2305	52.8	51.3	54.2	41	53.9	42.2	65.3
	Yes	2064	47.2	45.8	48.7	35	46.1	34.7	57.8
	Missing	9	-			0	-		
Gender	Boys	2235	51.1	49.6	52.5	33	43.4	32.3	55.3
	Girls	2143	48.9	47.5	50.4	43	56.6	44.7	67.7
	Missing	0	-			0	-		
gestational age (weeks)	≥37 weeks	4207	96.1	95.5	96.6	75	98.7	91.9	99.9
	<37 weeks	171	3.9	3.4	4.5	1	1.3	0.1	8.1
	Missing	0	-			0	-		
BMI before pregnancy	<25	3904	89.2	88.2	90.1	70	92.1	83	96.7
	≥25	473	10.8	9.9	11.8	6	7.9	3.3	17
	Missing	1	-			0	-		
Maternal age	<35 years	3086	70.5	69.1	71.8	60	78.9	67.8	87.1
	≥35 years	1292	29.5	28.2	30.9	16	21.1	12.9	32.2
	Missing	0	-			0	-		
Breast feeding	No	2655	60.8	59.3	62.2	45	59.2	47.3	70.2
	Yes	1712	39.2	37.8	40.7	31	40.8	29.8	52.7
	Missing	11	-			0	-		
Kindergarten	No	2252	52	50.5	53.5	30	41.7	30.4	53.9
	Yes	2075	48	46.5	49.5	42	58.3	46.1	69.6
	Missing	51	-			4	-		
Parity	Nulliparous	1756	40.7	39.2	42.2	27	36.5	25.8	48.5

	Multipara	2559	59.3	57.8	60.8	47	63.5	51.5	74.2
	Missing	63	-			2	-		
Season of blood test	March-May	950	21.7	20.5	23	19	25	16.1	36.5
	June-August	1376	31.4	30.1	32.8	28	36.8	26.3	48.7
	September-November	1327	30.3	29	31.7	22	28.9	19.4	40.6
	December-February	725	16.6	15.5	17.7	7	9.2	4.1	18.6
	Missing	0	-			0	-		
Z scores of BMI at 2 years	<2	3861	95	94.3	95.7	61	91	80.9	96.3
	≥2	202	5	4.3	5.7	6	9	3.7	19.1
	Missing	315	-			9	-		
Atopic dermatitis (3 y)	No	3794	86.7	85.6	87.6	36	90	75.4	96.7
	Yes	584	13.3	12.4	14.4	4	10	3.3	24.6
	Missing	0				36			
Serum 25-(OH)-D levels ng/mL	≥20 and <30	2238	51.1	49.6	52.6	18	50	34.5	65.5
	<20	1082	24.7	23.4	26	8	22.2	10.7	39.6
	≥30	1058	24.2	22.9	25.5	10	27.8	14.8	45.4
	Missing	0	-			40	-		

Table S2 Modified effect of maternal history of atopic dermatitis

	Maternal history of AD (-)	Maternal history of AD (+)
	Odds ratios (95% CI)	Odds ratios (95% CI)
Serum 25(OH)D concentrations		
Model 1		
<20 vs ≥30	1.13 (0.84-1.51)	0.91 (0.56-1.46)
≥20 and <30 vs ≥30	0.99 (0.76-1.28)	0.92 (0.61-1.39)
Model 2#		
<20 vs ≥30	1.03 (0.75-1.4)	0.82 (0.5-1.35)
≥20 and <30 vs ≥30	0.94 (0.72-1.22)	0.87 (0.57-1.31)
Deseasonalized 25(OH)D		
Model 1		
Deseasonalized 25-(OH)-D category 1 vs 3	0.93 (0.68-1.28)	0.81 (0.47-1.4)
Deseasonalized 25-(OH)-D category 2 vs 3	0.86 (0.63-1.19)	1.14 (0.67-1.96)
Deseasonalized 25-(OH)-D category 4 vs 3	0.9 (0.65-1.24)	0.89 (0.53-1.51)
Deseasonalized 25-(OH)-D category 5 vs 3	0.9 (0.66-1.24)	1.04 (0.6-1.8)
Model 2$		
Deseasonalized 25-(OH)-D category 1 vs 3	0.94 (0.68-1.3)	0.85 (0.49-1.47)
Deseasonalized 25-(OH)-D category 2 vs 3	0.87 (0.63-1.21)	1.23 (0.72-2.12)
Deseasonalized 25-(OH)-D category 4 vs 3	0.88 (0.64-1.22)	0.91 (0.53-1.54)
Deseasonalized 25-(OH)-D category 5 vs 3	0.9 (0.65-1.24)	1.06 (0.61-1.84)

AD: atopic dermatitis

Model 1 adjusted for gender and 25-(OH)-D / deseasonalized 25-(OH)-D

[#]Model 2 adjusted for all confounders listed in table S1 and an interaction term between maternal history of atopic dermatitis and 25-(OH)-D / deseasonalized 25-(OH)-D.
